# Prefrontal Cortex Dysfunction as a Precipitating Factor for Schizophrenia and Depression

**DOI:** 10.1111/jnc.70409

**Published:** 2026-03-10

**Authors:** Daniela L. Uliana, Anthony A. Grace

**Affiliations:** ^1^ Departments of Neuroscience, Psychiatry and Psychology University of Pittsburgh Pittsburgh Pennsylvania USA

**Keywords:** depression, dopamine, prefrontal cortex, schizophrenia

## Abstract

The prefrontal cortex (PFC) is critical for regulating stress responses through top‐down control over limbic and subcortical structures. The PFC undergoes a prolonged developmental process that only reaches maturation during adulthood, causing it to be highly sensitive to environmental insults during neurodevelopment, such as adolescence. During this critical period, synaptic pruning, the maturation of inhibitory GABAergic interneurons, and the refinement of dopaminergic transmission collectively establish the excitatory‐inhibitory balance necessary for adaptive behavior. Impairment of the PFC due to developmental disruptions increases susceptibility to maladaptive stress responses. These responses can, in turn, contribute to the development of major depressive disorder and schizophrenia. In depression, a dysfunctional PFC fails to effectively inhibit the amygdala, which contributes to hyperactivity in stress‐related circuits, hypodopaminergic states, and anhedonia. In schizophrenia, a neurodevelopmental PFC dysfunction would precipitate hippocampal circuit disruption driven by stress. The inability of an immature PFC to regulate the amygdala response to stress would trigger an increased excitatory drive to the ventral hippocampus, which is proposed to underlie the excessive limbic drive, hippocampal hyperactivity, and a hyperdopaminergic state. In addition, the activation of the mesocortical dopaminergic system by stress facilitates the PFC response to stress, both during adulthood and adolescence. A dopamine (DA)‐induced unregulated stress response disrupts the excitatory and inhibitory transmission within the PFC, which plays a critical role in its function. Understanding the interplay between stress and PFC activity/maturation to regulate the circuit toward adaptive or maladaptive outcomes offers critical insights for early intervention and prevention. Early changes in the PFC could underlie vulnerability to unregulated stress response and its consequent effect in contributing to schizophrenia and depression. In this way, early intervention may limit the impact and prevent further circuit dysregulation leading to pathological states.

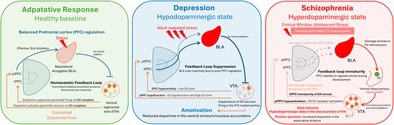

## Graphical abstract

Stress during adolescence or adulthood affects the ability of the prefrontal cortex (PFC) to adaptively regulate stress. Maladaptive adult stress leads to PFC hypofunction and a hypodopaminergic state in the medial, affect‐related ventral tegmental area (VTA), resulting in amotivation. Stress during adolescence triggers parvalbumin (PV) interneuron loss in the ventral hippocampus due to the inability of an immature PFC to regulate stress; this leads to a hyperdopaminergic state in the associative striatal‐projecting lateral VTA that is proposed to underlie positive symptoms. These reveal neurobiological trajectories for psychiatric disorders, pinpointing PFC‐amygdala circuit dysfunctions as potential targets for interventions.

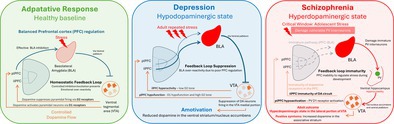

**Abbreviations**
BLA: basolateral amygdala
D1: dopamine receptor type 1
D2: dopamine receptor type 2
DA: dopamine
dACC: dorsal anterior cingulate cortex
GABA: gamma‐aminobutyric acid
GABA_A_
: GABA_A_ receptor
ilPFC: infralimbic prefrontal cortex
MAM: methylazoxymethanol acetate
mPFC: medial prefrontal cortex
PD: postnatal day
PFC: prefrontal cortex
plPFC: prelimbic prefrontal cortex
PNN: perineuronal nets
PV: parvalbumin interneurons
sgACC: subgenual anterior cingulate cortex
VTA: ventral tegmental area

## 1 Introduction

The prefrontal cortex (PFC) is a brain structure that plays a critical role in regulating executive functions (Friedman and Robbins 2022; Girotti et al. 2018) and, through its projections to numerous cortical and subcortical regions, can regulate diverse functions (Dembrow and Johnston 2014; Miller and Cohen 2001; Badre and Nee 2018). Thus, this area regulates responses such as decision‐making, working memory, and attention (Friedman and Robbins 2022; Miller and Cohen 2001). The PFC also regulates emotional responses by integrating information from multiple brain regions which, rather than reacting the same to all conditions, helps individuals adapt their behavior to specific situations (Kovner et al. 2019; Andrewes and Jenkins 2019). Significantly, the PFC is known to regulate responses to stress by exerting top‐down control over brain regions that generate emotional and physiological responses, such as the amygdala (Arnsten 2009; Etkin et al. 2011; Quirk and Beer 2006). Under normative conditions, the PFC can limit and redirect the response to stress‐induced fear and anxiety by suppressing excessive activation of the stress response systems (Quirk and Beer 2006). This allows individuals to respond effectively to challenges and triggers an adaptive response that maintains normal brain circuit function, and restores it to baseline when the threat has passed. However, in the face of chronic or severe stressful conditions, PFC function can be disrupted, which would decrease its inhibitory influence and lead to heightened emotional reactivity (Arnsten 2009; Liston et al. 2009; McKlveen et al. 2016). Therefore, the PFC is essential for maintaining balance between effective responses to stress, adaptive coping, and maladaptive stress responses.

Unregulated stress response due to PFC disruption can contribute dramatically to the emergence of psychiatric conditions, such as major depressive disorder and schizophrenia (Abé et al. 2021; Davey et al. 2008; McEwen and Morrison 2013; Caballero et al. 2016). The PFC has a key inhibitory control over the amygdala, which ultimately limits the magnitude and duration of excessive emotional reactivity in adaptive conditions (Quirk and Beer 2006; Rosenkranz and Grace 2002; Motzkin et al. 2015). Amygdala is a key area involved in emotional responses elicited by stress (Roozendaal et al. 2009; LeDoux 2000; Phelps and LeDoux 2005). Thus, dysfunction in the corticoamygdalar circuit, whether due to impaired development, structural abnormalities, or chronic stress exposure, may lead to maladaptive stress regulation. In major depressive disorder, there is a hyperactivation of the amygdala which contributes to impaired cognitive control over negative emotions that may leads to symptoms such as anhedonia (Drevets 2003; Sheline et al. 2001). Furthermore, disrupted PFC function in schizophrenia reduces the ability to filter and integrate information under stressful conditions, which exacerbates cognitive deficits and increases vulnerability to psychosis (Millan et al. 2016; Meyer‐Lindenberg et al. 2001; Goldman‐Rakic and Selemon 1997). Interestingly, both major depressive disorder and schizophrenia run in families and share genetic predisposition, PFC circuit dysfunction, and environmental risk factors (Cross‐Disorder Group of the Psychiatric Genomics Consortium 2013; Smeland et al. 2020). In fact, it has been demonstrated that clinical high‐risk individuals that do not transition to psychosis are suceptible to depression during adulthood (Salazar de Pablo et al. 2022; Lin, Wood, et al. 2015). One factor that seems to be determinant for the disorder's emergence is changes in PFC neurodevelopmental trajectories (Davey et al. 2008; Hoftman and Lewis 2011).

This intricate interplay between environmental factors, PFC dysfunction, and the emergence of psychiatric conditions has been explored extensively in preclinical models. These models enable a deeper understanding of the specific contributions of various factors to the behavioral and neurobiological domains associated with neuropathological states. The PFC in humans is more evolved and complex than in rodents, which may limit certain interpretations (Vertes et al. 2024). Nevertheless, the insights gained from rodents are valuable tools for understanding the circuitry associated with specific conditions. The medial PFC (mPFC) in rodents is typically divided into two regions, the prelimbic (plPFC) and infralimbic (ilPFC) regions (Vertes et al. 2024; Anastasiades and Carter 2021). These regions exhibit strong functional and connectivity properties similar to specific areas in the PFC of humans (Vertes et al. 2024; Anastasiades and Carter 2021). The rodent plPFC is regarded as the functional homolog of the human dorsal anterior cingulate cortex (dACC), which primarily corresponds to Brodmann area 32 (Bicks et al. 2015). Both rodents and humans' dACC have dense reciprocal connections with the hippocampus, amygdala, and nucleus accumbens/ventral striatum, which are critically involved in cognitive control and emotional regulation (Anastasiades and Carter 2021; Heilbronner and Hayden 2016). In contrast, the rodent ilPFC is considered analogous to the subgenual anterior cingulate cortex (sgACC), which corresponds to Brodmann area 25 (Vertes et al. 2024; Alexander et al. 2019; Heilbronner et al. 2016). These areas have been shown to have essential roles in affective state and fear response regulation (Scharnowski et al. 2020; Bush et al. 2022).

Understanding the neurodevelopment of the PFC is crucial to comprehending why this brain region emerges as a significant precipitating factor in stress‐related psychopathology (Caballero et al. 2016; Kolk and Rakic 2022). Adolescence marks a sensitive window in PFC neurodevelopment, during which extensive coordinated events contribute to an ideal excitatory‐inhibitory balance (Spear 2000; Chini and Hanganu‐Opatz 2021). These processes are essential for establishing the PFC's capacity to regulate limbic structures involved in emotional processing and stress reactivity based on experience (Yang and Tseng 2021). However, this prolonged maturation also makes the adolescent PFC highly vulnerable to environmental factors, such as stress (McEwen and Morrison 2013; Caballero et al. 2016). Disruptions during this developmental window can impair the formation of functional connectivity and inhibitory control, leading to persistent deficits in emotional regulation (Tottenham and Galván 2016). These alterations are strongly implicated in the emergence of psychiatric disorders, which often manifest clinically after adolescence but are rooted in earlier neurodevelopmental deviations (Shaw et al. 2010). Therefore, studying PFC maturation is not only essential to understanding its normative function but also to uncovering how early dysfunction predisposes individuals to lifelong vulnerability to mental illness.

## 2 Adolescence PFC Neurodevelopment

The PFC is one of the last brain areas to reach maturation (Giedd et al. 1999; Sowell et al. 1999; Spear 2000; Chini and Hanganu‐Opatz 2021). Early findings in humans demonstrated that cortical areas undergo a significant change in volume from late adolescence to adulthood (Sowell and Jernigan 1998). Gray matter in the PFC continues to increase throughout adolescence in humans (Gogtay et al. 2004). The process of increasing white matter content begins during childhood and continues into adulthood in the PFC (Giedd et al. 1999; Barnea‐Goraly et al. 2005). Neurons in the PFC start differentiating into the primary excitatory and inhibitory neurons during gestation, with maturation continuing throughout early postnatal periods (Kroon et al. 2019; Petanjek et al. 2008; Yang et al. 2014). PFC synaptogenesis begins during gestation and reaches its peak during early childhood (humans: 1–3 years old (Huttenlocher 1979; Huttenlocher and Dabholkar 1997; Selemon 2013; Lenroot and Giedd 2006)); rodents: postnatal day (PD) 15–30 (Van Eden and Uylings 1985; Caballero et al. 2016). Pruning occurs extensively during adolescence, around 10–20 years in humans and 30–40 days in rodents, where unnecessary synapses are removed (Van Eden and Uylings 1985; Caballero et al. 2016; Huttenlocher 1979; Huttenlocher and Dabholkar 1997; Selemon 2013). Pruning is a critical process that shapes the functional connectivity of the PFC, a process that is responsive to environmental factors (Kolb et al. 2012). Thus, the PFC undergoes a dynamic reorganization of activity and circuit structure during adolescence that is essential for establishing adult‐like prefrontal network properties and cognitive function (Pöpplau et al. 2024).

This delayed neurodevelopment can impact the ability of the PFC to regulate stress responses. Indeed, we have shown that juvenile rats at PD 21–30 are much more sensitive to the deleterious effects of stress than adults (Zhu and Grace 2022; Gomes and Grace 2017), and indeed respond to minor stressors in the same exaggerated manner as observed in adults with mPFC lesions (Uliana et al. 2021; Gomes and Grace 2017). Thus, while juveniles are in a very plastic stage of development where they are learning to effectively cope with their environment, they are also at a vulnerable stage with regard to trauma. Childhood trauma has been associated with a propensity to exhibit pathology in adults in terms of schizophrenia, depression, and drug abuse (Zhou, Sommer, et al. 2025; Dahoun et al. 2019; McLaughlin et al. 2019; Liu 2017). Therefore, at a stage when the mPFC has not yet fully developed, there is a lack of regulation over affective state and adversities.

The development of the PFC is likely influenced by signals originating from other subcortical structures, as they tend to mature earlier than the PFC (Caballero et al. 2016; Mills et al. 2014). In fact, amygdala fibers have already formed a bilaminar pattern in the mPFC around PD12‐16 in rats and continue to increase until adulthood (Cunningham et al. 2002; Verwer et al. 1996). The innervation of amygdala inputs is both onto pyramidal neurons and GABAergic interneurons (Cunningham et al. 2002, 2008; Gabbott et al. 2006). For instance, it's known that inputs from the amygdala to the PFC play a role in its excitability and likely maturation (Yang and Tseng 2021; Verwer et al. 1996; Johnson et al. 2016). The synaptic plasticity of amygdala inputs to PFC is known to reach adult levels by PD30, while the ventral hippocampus‐PFC pathway plasticity does not emerge until later into adolescence (PD50) (Caballero, Thomases, et al. 2014; Flores‐Barrera et al. 2014). This effect is regulated by different NMDA GluN2B subunits which likely mediate the contextual salient input selectively arising from the ventral hippocampus (Caballero, Thomases, et al. 2014; Flores‐Barrera et al. 2014). Thus, it's likely that the amygdala would be the main input regulating PFC excitability during earlier adolescence periods. In rodents, basolateral amygdala (BLA) inputs to the mPFC emerge earlier in life, around PD13‐30 (Bouwmeester, Wolterink, and van Wolterink 2002; Cunningham et al. 2002; Bouwmeester, Smits, and Van Ree 2002; Verwer et al. 1996; Pattwell et al. 2016). We have shown previously that the connectivity between the BLA and the PFC accelerates after adolescent stress exposure, which is correlated with BLA hyperexcitability in the short term after adolescent stress (Uliana et al. 2021). Therefore, it is possible that the maturation of the PFC is influenced by subcortical activity and external factors, either directly or indirectly.

Perhaps one of the central features of PFC maturation is the development of inhibitory GABAergic circuitry, especially the extensive maturation occurring during adolescence (Caballero, Thomases, et al. 2014; Caballero and Tseng 2016; Vandenberg et al. 2015). The mPFC undergoes coordinated changes in intrinsic and synaptic properties, including increased inhibitory input, reduced excitatory drive, and interneuron development, starting in early postnatal development (PD10‐20) (Kalemaki et al. 2022). In fact, GABA (gamma‐aminobutyric acid) is a critical component for establishing the balance of excitation and inhibition necessary for higher‐order cognitive functions (Caballero et al. 2021; Lam et al. 2022). Parvalbumin (PV)‐expressing interneurons are particularly important in this process due to their levels increasing during adolescence (Caballero, Flores‐Barrera, et al. 2014). The increased PV expression coincides with the increased excitatory inputs to the mPFC (Caballero et al. 2016; Caballero, Flores‐Barrera, et al. 2014). PV are fast‐spiking interneurons regulating the timing and synchrony of pyramidal neuron activity (Hu et al. 2014). PV expression increases significantly between P45 to PD55 along with the expression of its fast‐spiking properties (Caballero, Flores‐Barrera, et al. 2014; Drzewiecki et al. 2020). PV interneurons mature relatively late compared to other interneuron populations (Santos‐Silva et al. 2023), with their functional stabilization and maturation closely tied to the emergence of perineuronal nets (PNNs) which are specialized extracellular matrix structures that enwrap PV cells to lock in plasticity and protect them from insults (Balmer 2016; Cabungcal et al. 2013; Santos‐Silva et al. 2024). PNNs are known to develop around PD35‐50 (Caballero, Thomases, et al. 2014; Carceller et al. 2020). The maturation of PV interneurons and their PNNs is thought to mark the closure of critical periods for cortical plasticity (Santos‐Silva et al. 2024). Decreased PV excitability is hypothesized to contribute to the dysfunction of the PFC. It has been observed that suppressing PV during the juvenile period leads to adult sociability deficits that are similar to those observed in juvenile social isolation (Morishita 2021; Yamamuro et al. 2020).

Dopaminergic projections are known to target cortical areas via initially the medial portion of the ventral tegmental area (VTA) early on in life (embryonic in rodents and weeks after birth in humans) (Kalsbeek et al. 1990; Kalsbeek et al. 1988). The dopaminergic innervation continues to increase until adulthood in rodents (Kalsbeek et al. 1988). However, it is during adolescence that there is a marked surge in the organization and density of dopaminergic fibers, and increased dopamine (DA) receptor binding (Benes et al. 2000; Naneix et al. 2012; Tarazi and Baldessarini 2000). The maturation of dopaminergic transmission in the PFC parallels the evolution of behavioral flexibility, attention, motivation, and cognitive processes (Arnsten et al. 2015; Reynolds et al. 2018; Hauser et al. 2015; Saito et al. 2022; Van Duijvenvoorde et al. 2016); known to be undeveloped and markedly plastic during adolescence (Casey et al. 2008). Moreover, deficits in the dopaminergic pathways are known to contribute to various psychiatric disorders (Money and Stanwood 2013).

The DA influence over PFC activity would be age‐dependent due to the extended development of the DA system during adolescence (Leslie et al. 1991). Specifically, the DA type 1 receptor (D1) activation in the PFC at the adult stage induces the persistent depolarization plateau of pyramidal neurons via NMDA and post‐synaptic calcium recruitment; an effect that emerges at PD45 (Tseng 2004). Thus, the later D1 receptor expression emergence plays an important role in the facilitation of NMDA transmission and the adult phenotype (Tseng 2004; Flores‐Barrera et al. 2014). Interestingly, D1 receptors exert similar effects in facilitating GABAergic transmission and are the main regulator of DA mediating inhbition in the PFC at peripubertal stages PD15‐35 (Gorelova et al. 2002; Tseng et al. 2006), while DA inhibitory receptors, such as DA type 2 (D2) and type 4, mediated excitatory action on GABAergic transmission emerges only during late adolescence (~PD50) in PFC (Tseng et al. 2006; Tseng and O'Donnell 2006). Thus, the adult role of DA in facilitating GABAergic transmission is only achieved later in adolescence when both D1 and D2 receptors are expressed in the PFC. In this way, the DA input to the PFC plays a major regulatory role, particularly as it relates to stress. In the adult, stress is known to activate the mesocortical DA system (Baik 2020; Jackson and Moghaddam 2004), which through D1 excitation of pyramidal neurons and D2 inhibition of interneurons leads to PFC activation. This activation in turn suppresses stress responses via inhibition of amygdala activity. Therefore, when the DA input is not mature or is damaged, the ability of the PFC to limit stress responses is compromised. As a result, early in life it is expected that the inability to modulate the excitatory drive within the PFC by GABA mechanisms would further exacerbate vulnerability to insults in the PFC.

Other forms of neurotransmission in the PFC also mature during adolescence, such as serotoninergic, noradrenergic, and cholinergic signaling (Kolk and Rakic 2022; Garcia‐Garcia et al. 2017; Ramanathan et al. 2015). The serotoninergic and noradrenergic projections to the PFC occur early in life and are suggested to play a role in neuronal proliferation, migration, and differentiation (Janusonis et al. 2004; Garcia et al. 2019; Felten et al. 1982; Parnavelas and Blue 1982). The noradrenergic system is also proposed to play a role in the development of dopaminergic projections to the PFC, as it may provide a DA mechanism for reuptake via noradrenaline transporter due to the low levels of DA transporter on DA terminals in this region (Morón et al. 2002; Sesack et al. 1998). Noradrenergic transmission also appears to be important for GABA signaling in the PFC during neurodevelopment (Bortel et al. 2008). Acetylcholine has been demonstrated to innervate the plPFC in rodents during the first postnatal week, specifically targeting GABAergic interneurons (Janiesch et al. 2011). In addition, cholinergic signaling appears to also influence the development of functional interactions between the PFC and hippocampus, suggesting that these inputs may contribute to the emergence of coordinated prefrontal‐hippocampal network activity (Janiesch et al. 2011). Thus, acetylcholine may play a crucial role in the development of the PFC and its network. Therefore, these specific neurotransmitters likely play a crucial role in providing support and inputs to the dopaminergic and GABAergic systems.

As outlined above, the PFC has an extended time of maturation, which means its associated behavioral regulation is also plastic across this period. The PFC is crucial for cognitive and executive control, and changes in cognitive regulation accompany its maturation (Tsujimoto 2008). It has been demonstrated that in adolescence, rodents show a weaker performance on PFC‐dependent cognitive tasks and reduced PFC synaptic plasticity compared to adults, suggesting immaturity of synaptic plasticity and executive function in the PFC during adolescence (Konstantoudaki et al. 2018). Cognitive abilities are also associated with experience across neurodevelopment, as is PFC maturation. Thus, environmental factors play an important role in shaping the PFC maturation to allow more effective levels of plasticity that would contribute to optimize PFC‐regulated behaviors (Baker et al. 2025; Mackey et al. 2013). However, maladaptive adversities during its maturation are known to be a risk factor for psychiatric disorders, including disorders expressed later in adult life.

## 3 PFC Dysfuction in Depression

Major depressive disorder and schizophrenia are debilitating disorders that share multiple neurobiological mechanism (Xie et al. 2025; Reddy‐Thootkur et al. 2022; Adell 2020; Nusslock et al. 2025; Cross‐Disorder Group of the Psychiatric Genomics Consortium 2013). Major depressive disorder is a disorder characterized by a wide range of symptoms, including persistent depressed mood, anhedonia, amotivation, and fatigue that persist for 2 weeks or more (American Psychiatric Association 2013). PFC dysfunction has been reported in individuals with depression (Rogers et al. 2004; Drevets, Price, and Furey 2008) and stress is a significant external factor that can precipitate the onset of depression (Richter‐Levin and Xu 2018). In preclinical research, animal models used to study depression‐related symptoms often employ stress, including physical, emotional, and psychosocial, as a precipitating factor, leading to behavioral and neurobiological dysfunction consistent with depression in humans (Czéh et al. 2016; Muir et al. 2018). In fact, the mPFC is a crucial brain region that plays a vital role in regulating stress (McEwen and Morrison 2013). Thus, an inability of the mPFC to control or limit the response to stressful conditions may represent a factor in the development of depression (Liu et al. 2018; Murray et al. 2011).

The ability of mPFC to regulate amygdala activity may represent a critical circuit underlying depression emergence (Rosenkranz and Grace 2002; Motzkin et al. 2015). Amygdala hyperactivity and hyperresponsivity to negative valence stimuli are another neurobiological feature of depression (Murray et al. 2011; Doerig et al. 2016; Klug et al. 2024; Boukezzi et al. 2022). In humans, the dACC is known to attenuate emotional responses mediated by the amygdala once the threat is mitigated (Hariri et al. 2003). Indeed, this dysfunction is related to amygdala hyperactivity in patients with depression (Murray et al. 2011). It has been reported that individuals with cognitive vulnerability to depression have dACC hypoactivation leading to abnormal amygdala activation in response to emotional stimuli (Zhong et al. 2011). The plPFC targets GABAergic neurons in the BLA (Rosenkranz and Grace 2002), which decrease amygdala responsivity to stressors (Radley et al. 2006; Rosenkranz and Grace 2002; Jones et al. 2011; Figueiredo et al. 2003). These data suggest that the PFC‐amygdala pathway is involved in neurobiological changes in depressed patients and that PFC dysfunction may negatively impact the amygdala responsivity to stress, which in turn can lead to maladaptive behaviors.

Along with the apparent hypofunction of dACC and plPFC in depression and animal models, respectively, there is also a dysfunction in the ilPFC/sgACC (Mayberg et al. 1999; Mayberg et al. 2005; Drevets, Savitz, and Trimble 2008; Moreines et al. 2017). ilPFC send excitatory projections to BLA (Likhtik et al. 2006) which is proposed to mediate anxiety‐like behaviors (Radley et al. 2006). The ilPFC in rodents and sgACC in humans express an increased pattern of activity in models of depression/depression in humans (Mayberg et al. 1999; Mayberg et al. 2005; Drevets, Savitz, and Trimble 2008; Moreines et al. 2017). This hyperactivity is suggested to be due to a decreased inhibitory control in the sgACC, which is proposed to be caused by a reduction in GABAergic interneuron expression and function (Tripp et al. 2012). Indeed, GABA levels in this area in adolescents with major depressive disorder are decreased, which was correlated with anhedonia scores (Gabbay et al. 2012). One dysfunction that has been linked with PFC damage in response to stress are the somatostatin‐containing GABAergic interneurons (Fogaça and Duman 2019; Girgenti et al. 2019). Disruption of this local GABAergic circuit is believed to underlie the hyperactivity in the ilPFC which would be driving susceptibility to depression. Therefore, PFC‐related circuit mechanism in depression is proposed to be a product of plPFC hypofunction and ilPFC hyperfunction as a precipitator of amygdala hyperactivation.

PFC dysfunction also has been associated with cognitive impairment in depression (Pizzagalli and Roberts 2022). The sgACC contributes to cognitive deficits through emotional dysregulation. It exhibits heightened reactivity to negative‐valenced stimuli while blunting its responsiveness to positive‐valenced stimuli, similar to what is observed in patients with depression (Wallis et al. 2017; Alexander et al. 2023). sgACC hyperactivity is known to disrupt the balance between emotional and cognitive processing given that this area has strong connectivity with the default mode network (Zhou, Zhang, et al. 2025; Zhou et al. 2024). In depression, the sgACC exhibits hypoconnectivity with the posterior default mode network, such as the posterior cingulate cortex. This hypoconnectivity is associated with maladaptive rumination (Peng et al. 2021; Miller et al. 2015). The dysfunction in the dACC appears to contribute to cognitive deficits through disrupted cognitive regulation and behavioral generalization. In depression, the dACC has reduced connectivity with areas involved in cognitive control and underactivation during executive function tasks (Zhou et al. 2024; Miller et al. 2015). The dorsolateral PFC‐dACC‐sgACC circuit seems to integrate cognition and emotion. dACC receives input from deep dorsolateral PFC and projects to most of the sgACC layers. It is proposed to reduce sgACC output through parvalbumin inhibitory neurons (Joyce et al. 2020). This pathway allows cognitive control regions to flexibly regulate emotional output, and its disruption may explain the co‐occurrence of cognitive deficits and emotional dysregulation in depression (Myers et al. 2025). Therefore, the PFC circuit regulating emotional states seems to play a crucial role in the cognitive impairment observed in depression.

Another important behavioral domain associated with depression is amotivational states, such as anhedonia and helplessness, which has been associated with decreased activity of the DA system (Nestler and Carlezon 2006; Belujon and Grace 2017). For example, animal models of learned helplesness (Belujon and Grace 2014), chronic mild stress (Chang and Grace 2014; Moreines et al. 2017), and cold stress (Moore et al. 2001) models for the study of depression demonstrate a hypodopaminergic state with respect to the activity VTA DA neurons (Valenti et al. 2012; Chang and Grace 2014; Moreines et al. 2017; Rincón‐Cortés and Grace 2017; Douma and de Kloet 2019; Kaufling 2019). Also, inhibition of VTA DA neuron activity induces a depression‐like phenotype (Tye et al. 2013). The hypodopaminergic state in the VTA is believed to be driven by increased excitatory outputs of the BLA to the ventral pallidum (Chang and Grace 2014), as the BLA provides a direct input to the ventral pallidum (Novejarque 2011; Maslowski‐Cobuzzi and Napier 1994). Another pathway associated with an amotivational state is the ventral hippocampus connectivity with the nucleus accumbens. Disruption in the long‐term potentiation plasticity of this pathway is linked to anhedonia and helplessness behavior (Belujon and Grace 2014; LeGates et al. 2018). The disruption in hippocampal‐accumbens connectivity may stem from the involvement of the ilPFC via the thalamic nucleus reuniens (Zimmerman and Grace 2018), which serves as a crucial relay connecting the PFC with the hippocampus (Vertes et al. 2007). Indeed, inactivation of reuniens can partially restore the impaired plasticity of the ventral hippocampus and nucleus accumbens after repeated stress (Uliana, Gomes, and Grace 2022), which is known to induce a hypodopaminergic state. Furthermore, reuniens inactivation was able to reverse the decreased DA activity in the VTA and alleviate despair behavior in the forced‐swim test (Uliana, Gomes, and Grace 2022). Therefore, the hypodopaminergic state in the depression context may be a product of a circuit‐wide dysfunction upstream of the VTA.

**FIGURE 1:**
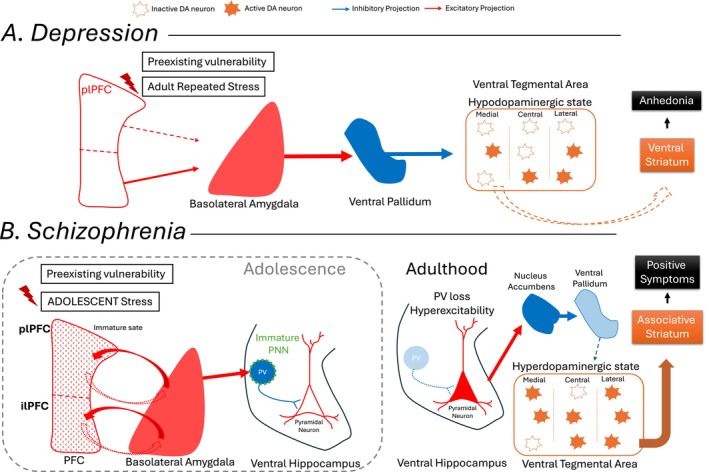
PFC‐circuit dysfunction associated with depression and schizophrenia. (A) A preexisting vulnerability to PFC dysfunction and/or a maladaptive response to stress, typically during adulthood, would lead to reduced function in the plPFC and increased activity in the ilPFC. This combination fails to inhibit the activity of the amygdala. Amygdala hyperexcitability drives increased activity in the ventral pallidum, which in turn provides inhibitory inputs to the VTA. Consequently, there is a decrease in the number of active dopamine neurons per track, particularly in the medial portion of the VTA, which decreases its outputs to the ventral striatum. This decrease is proposed to underlie the anhedonia and amotivation states. (B) During adolescence, the PFC is still undergoing maturation and exhibits limited capacity to regulate stress responses effectively. This immaturity contributes to increased amygdala excitability, which will disrupt PFC function through its excessive activation by stress. As a consequence, the PV‐expressing interneurons in the ventral hippocampus become particularly vulnerable due to the PNN immaturity to properly protect PV. In adulthood, the PV interneuron loss leads to ventral hippocampal hyperactivity; a well‐known domain observed in several animal models of schizophrenia. The resulting overactivation of excitatory projections from the ventral hippocampus to the nucleus accumbens enhances its output, which in turn increases inhibition of the ventral pallidum. The reduced ventral pallidal activity fails to suppress dopaminergic activity in the VTA, which leads to an increase in the number of spontaneously active dopamine neurons within the lateral VTA. This VTA projection in the lateral portion ultimately enhances dopaminergic transmission in the associative striatum, a mechanism thought to underlie the positive symptoms in schizophrenia. ilPFC, infralimbic portion of prefrontal cortex; PFC, prefrontal cortex; plPFC, prelimbic portion of prefrontal cortex; PV, parvalbumin; VTA, ventral tegmental area.

The activity of DA neurons in animal models of depression is associated with decreased activity in the medial and central portions of the VTA, which project to the mPFC, amygdala, and reward‐related nucleus accumbens shell (Figure 1A). In contrast, DA neurons in the lateral VTA project to the nucleus accumbens core and the associative striatum, which are more closely linked to schizophrenia neuropathology (Figure 1B) (Ikemoto 2007; Lammel et al. 2008). Thus, the dysregulation in DA function within the medial and central VTA is proposed to contribute to amotivational and anhedonic states (Belujon and Grace 2017; Ikemoto 2007). In addition, previous studies from our lab demonstrate that plPFC and ilPFC have opposite effects in regulating DA activity in the VTA. Inactivation of the plPFC decreases the number of active DA neurons in the VTA (Patton et al. 2013). Interestingly, inactivation of ilPFC increases VTA DA activity and activation decreases it (Patton et al. 2013). The experimental activation of ilPFC is consistent with the hypodopaminergic state in the VTA observed in animal models of depression (Belujon and Grace 2017). Also, the increased DA activity driven by ilPFC inactivation is mediated by ventral hippocampal subiculum and decreased DA VTA activity after ilPFC activation is regulated by BLA (Patton et al. 2013). Therefore, amygdala regulation by ilPFC seems to be a central node in depression. A combined action of plPFC inability to decrease amygdala activity and ilPFC hyperexcitability driving amygdala hyperexcitation are known to contribute to the hypodopaminergic state and behavioral negative affect in models of depression (Figure 1A).

The preexisting neurodevelopmental vulnerability within the PFC in controlling adversities is proposed to also underlie amotivational behaviors (McLaughlin et al. 2019; Uliana et al. 2020). We have demonstrated before that plPFC lesion during adolescence increases the susceptibility to helplessness and hypodopaminergic state later in adulthood (Uliana et al. 2020). We attribute this to the plPFC's inability to regulate amygdala function during adolescence, resulting in heightened susceptibility to amotivational states later in life. This aligns with the idea that adolescence is a critical period when the PFC establishes optimal connectivity with other brain areas (Caballero and Tseng 2016; Caballero et al. 2016; Zimmermann et al. 2019). In addition, we also observed that the effect of plPFC lesion in increasing subsequent vulnerability to helplessness is restricted to adolescence, as a plPFC lesion during adulthood does not impact the vulnerability to this condition (Uliana et al. 2020). The preexisting PFC dysfunction during development can be caused by an interaction of genetic and environmental factors that may lead to a disorder due to an unregulated stress response. For example, prenatal and perinatal adversities can disrupt PFC development (Schmitt et al. 2014) which would leave individuals susceptible to later unregulated stress response by PFC. The genetic risk may also be linked to early PFC dysfunction, as evidenced by genes associated with dendritic remodeling (Parker et al. 2020), and epigenetic mechanisms mediated by gene environment interactions (Hoffmann et al. 2017). Therefore, we propose that this preexisting PFC vulnerability arose from a combination of genetic and environmental factors, with genes not necessarily causing the disorder but predisposing elements to the deleterious effect of the environment.

The previous discussion shows that the inability of the mPFC to regulate stress will make an individual more vulnerable to depression in adulthood. However, when a plPFC lesion is combined with stressors during adolescence, the pathophysiological outcome in adulthood is more closely related to schizophrenia (Gomes and Grace 2017). We have demonstrated that adolescent rats with lesions in the plPFC exposed to mild stress conditions during the period of PD31‐40 exhibit increased DA activity in the associative striatal projecting lateral VTA, anxiety, cognitive deficits, and hyperlocomotion induced by amphetamine as adults. These effects parallel the exposure of a more intense stress protocol during the same adolescence period but without the plPFC lesion (Gomes and Grace 2017). We propose that the plPFC's neurodevelopmental dysfunction prevents the circuit from effectively managing milder stressful conditions, leading to a significant impact on the circuitry that mirrors the effects of more intense adversities. However, when only the plPFC lesion is carried out, the animal does not demonstrate these changes in domains that are more related to schizophrenia; instead, it turns out to be more vulnerable to depression during adulthood. Therefore, a preexisting PFC dysfunction during neurodevelopment should increase the vulnerability to psychiatric conditions. The environmental factors during this period are likely to determine the later outcome. In either case, the plPFC dysfunction appears to be a precipitating factor for different neuropathological conditions, especially during adolescence.

As mentioned previously, depression and schizophrenia run in families and share genetic predisposition (Cross‐Disorder Group of the Psychiatric Genomics Consortium 2013; Smeland et al. 2020). In fact, neurodevelopmental dysfunction in the PFC appears to be a shared mechanism across these disorders (Paus et al. 2008; Price and Drevets 2010; Hoftman and Lewis 2011). Although distinct PFC circuits and systems may contribute to the emergence of specific disorders, we propose that the most significant influence of PFC activity lies in its overall ability to regulate the stress response. When this regulatory function is compromised, dysregulated stress responses can impact subcortical regions (McEwen and Morrison 2013; Quirk and Beer 2006). These regions, depending on their neurodevelopmental trajectories, may undergo distinct alterations associated with specific disorders. For example, excessive stress during adolescence may lead to amygdala hyperactivation (Tottenham and Galván 2016; McLaughlin et al. 2019), which in turn increases excitatory projections to the ventral hippocampus (Janak and Tye 2015). Given the immaturity of local inhibitory circuits during adolescence, such hyperactivation would lead to permanent impairment in parvalbumin interneuron expression (Gomes et al. 2019). In contrast, PFC dysfunction not accompanied by extensive stress exposure may not trigger this severe cascade of events. Instead, it could leave the system vulnerable to milder environmental stressors that, over time, produce alterations more closely associated with depression. In this scenario, subcortical regions involved in emotional regulation, such as the amygdala, may be affected without causing irreversible damage in still‐developing structures like the ventral hippocampus.

## 4 PFC Dysfuction in Schizophrenia

Schizophrenia, a neurodevelopmental disorder, is proposed to arise from a combination of genetic predisposition and environmental factors (van Os et al. 2010; Tsuang 2000). It is characterized by the occurrence of positive symptoms (e.g., hallucinations and delusions), negative symptoms (e.g., social withdrawal, amotivation, blunted emotion), and cognitive symptoms (e.g., attention deficits, impairments in working memory) (Jauhar et al. 2022). Stress experienced during neurodevelopment is a significant environmental factor that contributes to the emergence of schizophrenia. For instance, childhood adversities have been extensively documented as strong predictors of later psychosis (Zhou, Sommer, et al. 2025). In fact, adolescents, particularly those at high risk for psychosis, are highly vulnerable to stress (Patel et al. 2020; Georgiades et al. 2023). This unregulated stress response in vulnerable individuals could be related to a preexisting inability of the mPFC to regulate stress response during neurodevelopment (Gomes et al. 2019) in a manner analogous to that occurring with depression.

mPFC dysfunction has been associated with the pathophysiology of schizophrenia (Lewis et al. 2005). Patients with schizophrenia exhibit decreased GABAergic transmission, including low levels of GABA and PV reduction in the dACC and sgACC during the disease state (Santos‐Silva et al. 2023; Beasley and Reynolds 1997; Volk et al. 2016; Gonzalez‐Burgos et al. 2010). This may contribute to impaired gamma oscillations and cognitive dysfunction in schizophrenia (Xu and Wong 2018; Gonzalez‐Burgos et al. 2015). Although GABA transmission is decreased in schizophrenia, it may cause an impaired excitatory‐inhibitory balance that fails to properly activate the PFC circuit reactivity, ultimately leading to functional hypofrontality, as reported in patients with schizophrenia (Uliana et al. 2024; Liu et al. 2021). In fact, in the MAM (methylazoxymethanol acetate) neurodevelopmental model of schizophrenia, hypofrontality has been observed (Kaneko et al. 2017). Thinning of the PFC is also a feature in MAM rats along with increased neuronal density without neuron loss, which suggests cortical reorganization rather than neurodegeneration (Moore et al. 2006). A decrease in PFC dendritic spines and working memory deficits are observed in MAM animals during adulthood, which may be associated with disrupted PFC plasticity during juvenile periods (Xing et al. 2016). This model also exhibits PFC reductions in PV‐expressing interneurons (Lodge et al. 2009; He et al. 2024) and early NMDA receptor hypofunction (Gulchina et al. 2017). In addition, reduced inhibitory tone accompanied by increased excitation of pyramidal neurons in the PFC has been reported during both adolescence and adulthood, suggesting that early inhibitory dysfunction is a core feature of this model (He et al. 2024). These changes may contribute to disrupted neuronal synchronization and abnormal augmentation of synaptic plasticity present in MAM rats (Goto and Grace 2006). Collectively, these PFC abnormalities may underlie the cognitive impairments in the MAM model (Moore et al. 2006; Featherstone et al. 2007). However, we hypothesized that the most relevant role of the PFC in schizophrenia is its antecedent mPFC dysfunction. A decreased activity in the mPFC would likely be determinant to the onset of psychosis due to poor stress regulation during adolescence.

PFC dysfunction appears to be present early in the disease state (Phillips and Seidman 2008; Van Der Velde et al. 2015). This suggests that mPFC dysfunction precedes the conversion to psychosis and may serve as a biomarker. Thus, the PFC's failure to regulate even mild adversities results in increased and unregulated amygdala activation, particularly during adolescence. In the adolescent stress model, we observed an increased activity of the BLA shortly after stress (Uliana et al. 2021; Zhu and Grace 2022). The immaturity of the PFC during this period of neurodevelopment hinders its ability to effectively regulate and limit the stress response. Consequently, the BLA becomes more reactive, which in turn would further affect the activity of the PFC through their reciprocal connectivity (Uliana et al. 2021) (Figure 1B). Activation of the amygdala in adult rats leads to long‐term potentiation of the PFC PV interneuron pathway and long‐term depression of excitatory transmission in the PFC, which is not present in the juvenile period. However, if the animal is exposed to stress early in life, there is precocious development of the long‐term depression of the PFC caused by BLA activation (Uliana et al. 2021). Such a precocious development before maturation may allow the juvenile to cope with extreme stress from which they would normally be protected (Callaghan and Tottenham 2016). Consequently, the juvenile may miss out on the opportunity to learn from its environment before this alteration, preventing the emergence of more effective coping strategies as adults. The excessive amygdala drive to PFC could also be contributing to the enhanced pruning in the PFC suggested to occur during the emergence of schizophrenia (Keshavan et al. 1994). All of these plastic events occurring in response to adversities might represent a mechanism that is precociously engaged in an attempt to control outcomes. However, although this forced adaptive mechanism would be effective in the short term, it is also proposed that it would lead to disruption of stress and emotional control in adults (Zhu and Grace 2022; McLaughlin et al. 2019).

In addition, the higher state of amygdala excitability during adolescent stress would, via an overdrive of the amygdala‐hippocampal PV projection, cause a loss of hippocampal PV interneurons (Berretta et al. 2004) (Figure 1B). The loss is proposed to occur due to the immaturity of PNNs in the ventral hippocampus during the adolescent period, which leaves the PV interneurons vulnerable to excitotoxic damage and oxidative stress, leading to PV neuron loss (Santos‐Silva et al. 2023). It appears that this event is restricted to adolescence, as the PNNs during adulthood are better equipped to regulate and protect the PV interneurons (Santos‐Silva et al. 2024). The PV loss would be driving the sustained increase in excitability in the limbic hippocampus, which is proposed to be the primary pathological site of schizophrenia (Gomes et al. 2019). This hippocampal hyperactivity in turn drives the increased DA VTA activity via the accumbens‐ventral pallidum pathway (Lodge et al. 2009) (Figure 1B). The cognitive and negative symptoms are also suggested to originate from the ventral hippocampus projecting to the prefrontal cortex and amygdala, respectively (Grace and Uliana 2023). Early hippocampal disruption (e.g., after poor stress regulation) would further contribute to abnormal activity in the prefrontal network. This disruption is likely to underlie the long‐lasting disruption of hippocampus‐prefrontal communications observed in the disease state (Xu et al. 2021). Therefore, an early unregulated stress response would precede and drive the pathology observed in the later transition to schizophrenia.

The concept of an unregulated stress state during adolescence is also supported by a neurodevelopmental animal model of schizophrenia, the MAM model (Gomes et al. 2016). Offspring from pregnant rats injected with MAM during gestational Day 17 express a combination of behavioral and neurobiological changes related to schizophrenia, including the limbic hippocampus hyperexcitability (Gomes et al. 2016; Modinos et al. 2015; Moore et al. 2006). MAM rats have been shown to exhibit high levels of anxiety during adolescence and heightened sensitivity to stress (Du and Grace 2013). Additionally, they demonstrate increased BLA activity during adolescence (Du and Grace 2016a), suggesting an inefficient control by the mPFC. Indeed, treating the heightened stress response with diazepam or environmental enrichment during adolescence in MAM rats can normalize anxiety and prevent the transition to a hyperdopaminergic state later in adulthood (Du and Grace 2013; Du and Grace 2016a; Zhu and Grace 2021). This suggests that stress management during neurodevelopment in highly susceptible individuals could prevent the transition to psychosis. Cognitive behavioral therapy, for instance, is proposed to have some potential in preventing psychosis in high‐risk individuals, due to the lower stress reactivity after the treatment (Hardy et al. 2016). This therapeutic strategy has been demonstrated to reduce limbic reactivity in individuals with anxiety and mood disorders (Yuan et al. 2022; Bomyea et al. 2020; Månsson et al. 2016). Therefore, stress management could potentially contribute to the mPFC's regulation of amygdala reactivity during neurodevelopment, thereby preventing the ventral hippocampus dysfunction observed in schizophrenia.

The nucleus reuniens may also represent an additional neurobiological mechanism contributing to ventral hippocampus dysfunction in the context of schizophrenia, as it receives inputs from the PFC. In fact, thalamic hyperperfusion is present in the early stages of schizophrenia (Orlović et al. 2025) along with thalamic‐cortical hypoconnectivity that persists into chronic stages (Woodward and Heckers 2016). Inhibition of the ilPFC increases VTA DA activity via nucleus reuniens–ventral hippocampus pathway (Zimmerman and Grace 2016). Additionally, activation of the nucleus reuniens also leads to a hyperdopaminergic state through the ventral hippocampus (Zimmerman and Grace 2016). This suggests that a PFC dysfunction may be increasing the reuniens activity to elicit its impact on dopaminergic activity. Interesting, the ilPFC influences reuniens activity via direct projections and indirectly via the thalamic reticular nucleus (Zimmerman and Grace 2018). In MAM rats, there is a decrease in parvalbumin expression in the thalamic reticular nucleus, which could lead to a decreased inhibitory input to reuniens. Also, the ilPFC inhibition decreased the number of VTA DA neurons instead of increasing them as observed in the naïve condition (Zhu et al. 2021). This suggests changes in the ilPFC regulation of VTA activity in these conditions likely are due to the changes in the thalamic reticular nucleus. Under normal conditions, ilPFC inactivation would affect primarily the thalamic reticular nucleus‐mediated feedforward inhibition, which would disinhibit the reuniens‐ventral hippocampus pathway to increase dopaminergic activity. In contrast, in MAM rats with reduced parvalbumin in the thalamic reticular nucleus, this weakened feedforward inhibition allows direct ilPFC–reuniens glutamatergic inputs to dominate. Therefore, inhibiting the ilPFC would decrease the direct glutamatergic inputs to the reuniens in MAM rats, leading to a reduced activation of the reuniens‐ventral hippocampus and its consequent drive of a hyperdopaminergic state. The thalamic dysregulation could represent an additional pathway working in conjunction with the hyperexcitability of the amygdala, both arising from poor regulation of these circuits by the PFC.

Cognitive impairment also represents an early domain of dysfunction in the disease state and may emerge in parallel with abnormal development of the PFC. This may contribute to the onset of cognitive deficits prior to the development of psychosis (McCutcheon et al. 2023; Chini and Hanganu‐Opatz 2021; Pöpplau et al. 2024). Cognitive changes in schizophrenia involve reduced processing speed, working memory, attention, and cognitive control (McCutcheon et al. 2023; Tripathi et al. 2018). The dACC plays a role in the schizophrenia cognitive deficits by impairing conflict monitoring and error processing, while the sgACC may influence cognitive processes by changing functional connectivity patterns (Wang et al. 2015). Decreased dACC activation is observed during conflict and error commission on cognitive tasks in patients with schizophrenia (Kerns et al. 2005). Reduction in the dACC circuit during error commission is proposed to affect reinforcement‐based learning, as blunted dACC responses seem to be inversely related to error rates (Polli et al. 2008). PFC circuit thinning is suggested to be associated with decreased activation during cognitive processes (Schultz et al. 2012). Evidence from the MAM animals also supports this human finding, which indicates thinning of the PFC and cognitive deficits in MAM rats (Moore et al. 2006; Modinos et al. 2015; Mar et al. 2017). Therefore, cognitive changes due to an early PFC dysfunction could represent a biomarker for increased susceptibility to schizophrenia.

## 5 Interplay Between VTA DA and PFC

**FIGURE 2:**
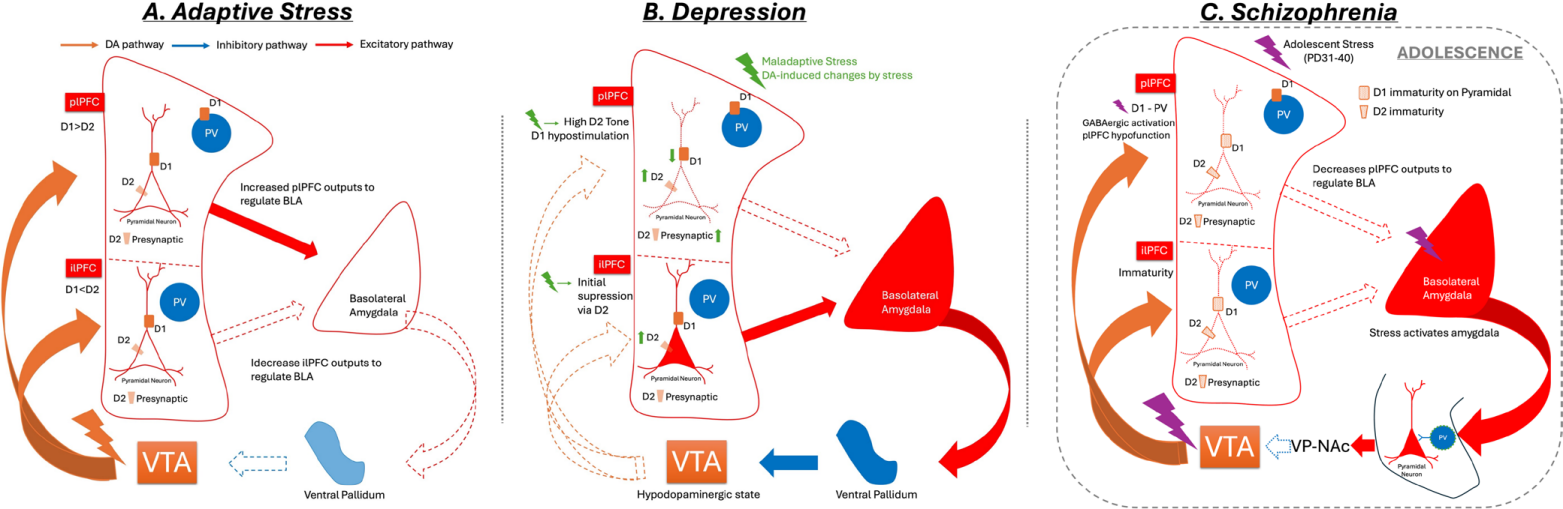
DA‐driven modulation of PFC contributing to adaptive and maladaptive stress response associated with depression and schizophrenia. (A) Under adaptive stress response in adulthood, stress will increase DA neuron activity in the VTA and lead to increased outputs to PFC. DA in the plPFC will activate this area via its DA action on D1 receptors present on pyramidal neurons and inhibit GABAergic interneurons via D1. In the ilPFC, DA will lead to suppression of pyramidal neurons firing via D2 activation. This would serve to decrease the stress‐induced amygdala activation, which limits amygdala ability to further influence VTA activity via ventral pallidum. (B) In depression, repeated maladaptive stress will lead to a D1 hypofunction and D2‐high tone in the plPFC that will reduce pyramidal neuron firing and ultimately decrease the plPFC activity. This plPFC hypofunction will fail to regulate amygdala hyperexcitability. An amygdala hyperactivation would decrease the dopaminergic activity in the VTA via ventral pallidum activation. Lower DA inputs to the plPFC will perpetuate the lack of excitatory drive of DA over the plPFC. In the ilPFC, an initial DA activation of the ilPFC would cause an initial suppression of pyramidal neuron activity via D2 receptor stimulation but as the stress continues, this would result in a prolonged unregulated stress response and DA depletion with its consequent increased excitability by the lack of D2 activation. (C) For schizophrenia, during the adolescent period, stress will lead to an increased DA activity in the VTA, and its outputs to the PFC. In the immature plPFC, DA will increase GABAergic activity via D1 activation in the PV interneurons, which will decrease the plPFC's ability to regulate stress‐induced amygdala excitability. Increased amygdala drive to the ventral hippocampus will damage PV interneurons due to the immaturity of the PNN. It will cause PV loss and allow the ventral hippocampus to be hyperexcitable that will drive the perpetuation of a hyperdopaminergic state in the VTA via the nucleus accumbens‐ventral pallidum pathway. ilPFC, infralimbic portion of prefrontal cortex; PFC, prefrontal cortex; plPFC, prelimbic portion of prefrontal cortex; PNN, perineuronal net; DA, dopamine; PV, parvalbumin; VTA, ventral tegmental area.

Under normal conditions in adulthood, stress will increase DA neuron activity in the VTA (Valenti et al. 2011) and activate the mesocortical pathway (Baik 2020; Jackson and Moghaddam 2004). Conserved across species, the majority of pyramidal neurons and GABAergic interneurons express both D1 and D2 receptors (Bouthenet et al. 1987; Gaspar et al. 1995; Santana et al. 2009; Muly et al. 1998). It has been demonstrated that the plPFC has a higher DA innervation than the ilPFC (Islam et al. 2021; Williams 1998). This likely plays a more pronounced role in the modulatory action of DA, especially during stress. The D1:D2 ratio function appears to be higher in the plPFC compared to a more balanced and likely D2‐biased mechanism in the ilPFC (Barker et al. 2013; Santana et al. 2009; Ball et al. 2022). Thus, the release of DA in the plPFC will activate this area via its DA action on D1 receptors present on pyramidal neurons and inhibit GABAergic interneurons via D1/D2 (Seamans and Yang 2004; Seamans et al. 2001). In the ilPFC, DA would instead be driving suppression of firing via D2 activation (Mueller et al. 2010). This would serve to put a brake on stress‐induced amygdala activation (Figure 2A), thus self‐limiting the impact of stress. Studies in humans have demonstrated that stress‐related tasks increase DA in the ACC (Ko et al. 2009; Arnsten et al. 2023; Nagano‐Saito et al. 2013), supporting the preclinical data in the mPFC. In humans, research has shown that a higher DA tone in the ACC is linked to improved emotional regulation and reduced amygdala reactivity (Berry et al. 2019; Kobiella et al. 2010). It is proposed that in uncontrollable stress situations, the top‐down control exerted by higher‐order PFC, such as the dACC, would be lost and flipped to a more emotional sgACC (Arnsten et al. 2023).

Acute stress is known to increase DA activity in the VTA (Valenti et al. 2011), which in turn would activate the plPFC and inhibit the ilPFC (Figure 2A), providing a feedback limitation of the stress response. However, repeated stress can lead to decreased DA activity in the VTA (Belujon and Grace 2017; Valenti et al. 2012; Moore et al. 2001; Chang and Grace 2014), which is associated with blunted motivation in individuals with depression (Belujon and Grace 2017). The acute increase in DA inputs to the plPFC would effectively increase its excitability to better modulate stress. But when abnormal and repeated levels of stress are present, they lead to a D1 dysfunction (Shinohara et al. 2018; Anderson et al. 2019) and likely a high D2 tone in plPFC, which would reduce the firing of pyramidal neurons (Figure 2B). This reduction in pyramidal neuron firing would then cause a loss of top‐down control over amygdala hyperactivity. An abnormal amygdala activation would also exacerbate the hypodopaminergic state in the VTA (Belujon and Grace 2017) (Figure 2B). Lower DA inputs to the plPFC would further perpetuate the lack of excitatory drive of DA over the plPFC that would normally limit the effects of repeated stress. The diminished DA activation of the ilPFC would lead to an initial suppression of activity via D2 receptor stimulation. As the stress continues, this would result in a prolonged unregulated stress response and DA depletion (Mizoguchi et al. 2000; Mizoguchi et al. 2008), which would allow it to increase excitability, as is often observed in animal models of amotivation (Belujon and Grace 2017; Arnsten et al. 2023; Mizoguchi et al. 2008). In fact, individuals with depression exhibit a reduction in DA input and lower stimulation of the D1 receptors in the dACC, which is known to contribute to its hypoactivity (Rao et al. 2019; Guo et al. 2003; Conn et al. 2020). These findings parallel the reduced D1 signaling and low DA release in the plPFC in stress‐based animal models of depression (Lin, Borders, et al. 2015; Matuszewich et al. 2014). Indeed, lower levels of DA transporter, tyrosine hydroxylase, and DA decarboxylase were reported in the sgACC in some populations of patients with depression (Gliaudelytė et al. 2025). Therefore, in the sgACC, deficits in dopaminergic input may fail to reduce its excessive activity, which in turn contributes to amygdala activation, leading to sustained limbic hyperactivity, heightened negative affect, and impaired emotional regulation in depression.

As repeated stress in adults appears to profoundly impact the DA‐related circuitry in the PFC that may ultimately contribute to the emergence of depression, it is important to note that the circuit reacts to stress differently during adolescence. This is because the PFC DA transmission is still undergoing maturation (Caballero et al. 2016; Reynolds et al. 2018). During adolescence, as the mesocortical pathway continues to develop and the expression of D1 and D2 increases in the mPFC, it is anticipated that the immaturity of the DA input would not effectively regulate the stress response. As discussed before, D1 action on pyramidal neurons emerges at PD45 (Tseng 2004) and D2 action on GABAergic around PD50 (Tseng et al. 2006; Tseng and O'Donnell 2006), which leaves the D1 action as the primary mechanism facilitating GABAergic transmission in the mPFC during PD15‐25 (Gorelova et al. 2002; Tseng et al. 2006). For instance, adolescent stress could lead to an increase in DA activity in the VTA, which in turn would enhance the output drive to the mPFC. During the adolescent stress period (PD31‐40), the increased DA inputs to the mPFC would lead to a GABAergic activation via D1 (Figure 2C). This activation could potentially prevent the mPFC from regulating stress by reducing the reactivity of the amygdala. This would further intensify amygdala hyperactivity, causing it to exert detrimental effects on PV interneurons in the ventral hippocampus during adolescence (Figure 2C). Thus, it is anticipated that the inability to regulate the excitatory drive within the PFC through GABA mechanisms mediated by the D2/D1 mechanism would exacerbate vulnerability to insults in the PFC.

While the proposed neurodevelopmental changes in PFC DA transmission are crucial for schizophrenia emergence, it appears that PFC DA transmission continues to contribute to the neuropathological state even after the transition to psychosis. It is described that patients with schizophrenia exhibit a reduction in the DA content in the PFC areas, including dACC and sgACC (Abi‐Dargham and Moore 2003; Frankle et al. 2022; Rao et al. 2019; Kambeitz et al. 2014). Decreased DA function in the dACC is proposed to contribute to the cognitive impairments observed in schizophrenia (Rao et al. 2019; Guo et al. 2003; Conn et al. 2020). Studies in humans demonstrate reduced D1 receptor binding, changes in tyrosine hydroxylase fiber distribution, and lower DA synthesis and release capacity in the dACC of individuals with schizophrenia (Rao et al. 2019; Frankle et al. 2022; Okubo et al. 1997; Benes et al. 1997; Lewis and Sweet 2009). In the sgACC, dysregulated DA transmission is also proposed to be the underlying cause of the blunted affect and deficits in regulation of emotion (Gliaudelytė et al. 2025). This neurobiological circuit is closely related to the negative symptoms present in schizophrenia (Nelson et al. 2015; Potkin et al. 2002; Chuang et al. 2014). Although a hyperdopaminergic state is associated with the core neurobiology of schizophrenia, the increased DA transmission originating from the VTA appears to be restricted to the lateral portion of the VTA in rodents (Gomes and Grace 2017; Ikemoto 2007; Uliana, Zhu, et al. 2022). This lateral portion projects to the associative striatum, where in humans there is a marked increase in the fluorodopa reuptake and amphetamine‐induced DA release in patients with schizophrenia (Howes et al. 2009; Abi‐Dargham et al. 2009). Therefore, the dynamics of DA transmission in schizophrenia demonstrate a poor regulatory role of DA in PFC areas, while also exhibiting an increased transmission in subcortical areas, such as the striatum.

## 6 Insights for Treatment and Prevention

Current treatments for schizophrenia with one exception target D2 receptors in the associative striatum (Meyer 2025). This target was discovered by serendipity rather than by rational targeting. As a result, while D2 antagonists may be effective at treating the DA‐mediated positive symptoms, they are fraught with side effects related to off‐target DA antagonism, such as amotivation, hyperprolactinemia, akathisia, and other pathophysiologies (Iwata et al. 2016; Siafis et al. 2023; Sakurai et al. 2013). Furthermore, these treatments do not substantially impact the other major symptom categories of schizophrenia, such as the negative or cognitive symptoms (Grace and Uliana 2023). Recent research has provided evidence as to why this is the case. Therefore, studies have shown that there is little evidence for a primary deficit in the DA system; alternately, it appears that the DA system is normal but is being dysregulated by other structures (Grace 2016). Primary among these is the hippocampus. Multiple studies have demonstrated that in schizophrenia, there is hyperactivity in the limbic anterior hippocampus that corresponds to positive symptom severity (McHugo et al. 2019; Heckers and Konradi 2015). Animal studies have shown that hyperactivity in the rodent ventral limbic hippocampus (homologous to the human anterior limbic hippocampus) will put the DA system in a hyper‐responsive state, causing an overactivation to both salient and nonsalient stimuli (Lodge and Grace 2007; Grace 2012). This is proposed to be the basis of the schizophrenia patient being unable to distinguish between salient and nonsalient stimuli, being overwhelmed and unable to focus on a single stimulus, and misinterpreting neutral stimuli as threatening (Kätzel et al. 2020; Kowalski et al. 2021); a condition referred to as aberrant salience (Kapur 2003). Furthermore, a hyperactive and dysrhythmic limbic hippocampus will also interfere with other hippocampal targets. Therefore, pathology within the hippocampal‐amygdala‐cingulate cortex pathway is proposed to lead to negative symptoms, and disruption of the hippocampal‐PFC pathway is proposed to lead to cognitive disruption (Grace and Uliana 2023; Uliana, Zhu, et al. 2022). What causes the hippocampus to be hyperactive and dysrhythmic? It is suggested that the pyramidal output neuron hyperactivity is driven by a loss of GABAergic PV‐containing hippocampal interneurons. Importantly, these neurons have been shown to be particularly vulnerable to stress during the juvenile period. To put this all together, when the mPFC fails to control the amygdala, the amygdala‐hippocampal pathway that innervates PV neurons is hyperactive, leading to excitotoxic damage to the interneurons, causing hippocampal pyramidal neurons to be hyperactive and interfering with normal rhythmic function (Figure 1B) (Gomes et al. 2019).

This raises the question about the most effective therapeutic intervention. One potential intervention would be to compensate for the deficit in PV interneuron number. This would require potentiation of the remaining GABAergic pyramidal neuron inhibition. In general, when it is necessary to potentiate GABAergic synapses, a benzodiazepine‐based positive allosteric modulator is the most effective strategy. While a broad‐spectrum benzodiazepines would have unwanted sedative properties by acting on different GABA_A_ receptor subtypes (de la Iglesia‐Larrad et al. 2020), the hippocampal pyramidal neuron targets of the PV interneurons are enriched with the GABA_A_ alpha 5 subunit (Fritschy and Panzanelli 2014; Heldt and Ressler 2007; Ramos et al. 2004; Serwanski et al. 2006). By utilizing a benzodiazepine‐like compound that is selective for potentiating GABA_A_ at alpha 5 containing receptors, one could selectively potentiate the inhibitory influence on pyramidal neurons. Indeed, in preclinical work on MAM rats, we found that a selective GABA_A_ alpha 5 positive allosteric modulator was highly effective in normalizing hippocampal hyperactivity and restoring DA system function to normal (Gill et al. 2011; Uliana et al. 2026). Another approach would be to decrease excitability of the hippocampal pyramidal neurons directly. A new compound, evenamide, is a sodium channel blocker that selectively attenuates hyperactivity in pyramidal neurons (Uliana et al. 2025). This drug was found to be effective in clinical trials (Anand et al. 2023, 2025) and in our hands also normalized hippocampal hyperactivity while also normalizing DA system activity and reversing cognitive and social negative symptoms (Uliana et al. 2025). Finally, a drug that has recently been introduced for the treatment of schizophrenia, KarXT, was found to be effective for schizophrenia without directly impacting the DA receptors (Mohammed et al. 2025; Azargoonjahromi 2024). While the precise site of action has not been definitively shown, it is likely that this compound may impact the hippocampus directly, or act at downstream targets of the hippocampus. In summary, targeting the site of pathology related to hippocampal hyperactivity would be a more effective treatment strategy than current D2 antagonist administration with respect to greater efficacy and less side‐effect potential (Grace and Uliana 2023).

The previous section demonstrated how we may base a more effective therapeutic approach by targeting the site of dysfunction in the schizophrenia brain. However, these are interventions that compensate for an already‐disrupted system driven by neuronal loss. A more effective approach would be to prevent the transition to schizophrenia in susceptible individuals. In fact, PFC disruption could also represent a biomarker of susceptibility to schizophrenia. Individuals at clinical high risk for psychosis show disrupted resting‐state thalamo‐cortical functional connectivity and faster gray matter reduction in the PFC, which may predict psychosis conversion (Cannon 2016). Indeed, vulnerability to psychosis is associated with PFC activation abnormalities during cognitive tasks (Fusar‐Poli et al. 2007). Thus, early changes in PFC parameters could be an indicator of disease vulnerability that would open a window for intervention to decrease conversion to schizophrenia. Given our argument above regarding PFC inability to regulate stress responses as a pathophysiological determinant in the onset of schizophrenia, one potential means to circumvent the transition to schizophrenia would be to decrease the impact of stress in susceptible individuals. Indeed, in preclinical studies of MAM rats, we found that administering the anti‐anxiety agent diazepam in the peripubertal period was sufficient to prevent the hippocampal PV loss and the resultant hyperdopaminergic state (Du and Grace 2013, 2016a, 2016b). As a proof of principle, another compound known to be anxiolytic, the mGluR2/3 agonist pomaglumetad, was also found to prevent this transition (Sonnenschein and Grace 2020). Therefore, treating at‐risk individuals based on family history of schizophrenia or abnormally augmented stress responses may be an effective strategy. Of course, such a strong pharmacological intervention would not be ideal given that young children would be treated, the majority of whom would not transition to psychosis. Given this, we also found that a psychosocial type of antianxiety intervention, which in rodents consisted of enriched environment and augmented social contact, was also effective in preventing MAM‐induced transition to a psychosis‐like state (Zhu and Grace 2021).

PFC dysfunction early on also is associated with depression vulnerability. For example, less positive coupling between the sgACC and dorsolateral PFC during childhood predicted increases in internalizing behaviors (anxiety and depression) by 11 years of age (Whitfield‐Gabrieli et al. 2020). This pattern was associated with familial risk for major depressive disorder (Whitfield‐Gabrieli et al. 2020). In addition, as mentioned earlier, schizophrenia and depression tend to run in the same families. In individuals that are at ultra‐high risk for schizophrenia based on attenuated psychotic signs, those that do not transition are more susceptible to affective disorders as adults (Salazar de Pablo et al. 2022; Lin, Wood, et al. 2015). This demonstrates that both disorders share risk factors, such as PFC dysfunction, and that early identification and intervention may help prevent the transition for these disorders. This is further substantiated in animal models, in which MAM rats treated with diazepam to prevent the onset of a psychosis‐like state are much more vulnerable to stress‐induced depression as adults (Du and Grace 2013). Therefore, it is likely that both schizophrenia and depression may be linked to disrupted regulation of stress responses, with the differentiating factor being strong stress exposure during adolescence leading to schizophrenia. However, if they are protected from this early stress, they will nonetheless be susceptible to affective disorders later in life. As a result, controlling the antecedents to the exaggerated stress response may be an effective means to prevent the onset of multiple psychiatric conditions.

## 7 Conclusion

The evidence reviewed here supports a model in which prolonged neurodevelopment of the PFC renders stress‐regulatory circuits particularly vulnerable during adolescence. This would have lasting consequences for limbic, hippocampal, and dopaminergic function. Disruption of PFC‐mediated stress regulation, due to impaired maturation, genetic susceptibility, or environmental adversity, may cause the system to be vulnerable to circuit dysfunction related to depression or schizophrenia. Unregulated stress responses during development may represent a shared antecedent of multiple psychiatric disorders and underscore the importance of early interventions that restore circuit balance. Stress timing and intensity, along with preexisting PFC dysfunction, would be the determining factors for a specific neuropathological condition. However, future studies are needed to delineate the specific neurobiological circuits underlying PFC dysregulation, with particular emphasis on thalamic mechanisms alongside the well‐characterized amygdalar connectivity that together disrupt PFC–hippocampal communication under stress. Investigating sex‐specific trajectories of stress responsivity and PFC regulation is also essential, as these differences likely shape vulnerability, symptom expression and disease timing. Notably, such mechanisms remain unexplored in mechanistic models despite clear sex differences in susceptibility to schizophrenia and depression. Studying neurodevelopmental PFC function in these contexts is crucial to understanding the neuropathological emergence and potentially gathering insights into biomarkers and preventive interventions before irreversible circuit remodeling occurs.

## Author Contributions

**Daniela L. Uliana:** conceptualization, writing – original draft. **Anthony A. Grace:** conceptualization, project administration, supervision, writing – review and editing.

## Funding

This work was supported by the National Institute of Mental Health, MH57440 to Anthony A. Grace.

## Conflicts of Interest

Anthony A. Grace has received consulting fees from Alkermes, Lundbeck, Takeda, Roche, Lyra, Concert, and research funding from Lundbeck, Newron, and Merck. Daniela L. Uliana declare no conflicts of interest.

## Data Availability

The authors have nothing to report.
